# Investigation of Highly Active Carbon‐, Cobalt‐, and Noble Metal‐Free MnO_2_/NiO/Ni‐Based Bifunctional Air Electrodes for Metal–Air Batteries with an Alkaline Electrolyte

**DOI:** 10.1002/gch2.202200223

**Published:** 2023-04-07

**Authors:** Marvin Kosin, Simon Dondrup, Jan Girschik, Jens Burfeind, Ulf‐Peter Apfel, Anna Grevé

**Affiliations:** ^1^ Fraunhofer Institute for Environmental Safety and Energy Technology UMSICHT Osterfelder Str. 3 46047 Oberhausen Germany; ^2^ Inorganic Chemistry I Faculty of Chemistry and Biochemistry Ruhr University Bochum Universitätsstr.150 44801 Bochum Germany

**Keywords:** air electrodes, bifunctional, metal–air batteries, noble metal free, oxygen electrodes

## Abstract

Compared to other battery technologies, metal–air batteries offer high specific capacities because the active material at the cathode side is supplied by ambient atmosphere. To secure and further extend this advantage, the development of highly active and stable bifunctional air electrodes is currently the main challenge that needs to be resolved. Herein, a highly active carbon‐, cobalt‐, and noble‐metal‐free MnO_2_/NiO‐based bifunctional air electrode is presented for metal–air batteries in alkaline electrolytes. Notably, while electrodes without MnO_2_ reveal stable current densities over 100 cyclic voltammetry cycles, MnO_2_ containing samples show a superior initial activity and an elevated open circuit potential. Along this line, the partial substitution of MnO_2_ by NiO drastically increases the cycling stability of the electrode. X‐ray diffractograms, scanning electron microscopy images, and energy‐dispersive X‐ray spectra are obtained before and after cycling to investigate structural changes of the hot‐pressed electrodes. XRD results suggest that MnO_2_ is dissolved or transformed into an amorphous phase during cycling. Furthermore, SEM micrographs show that the porous structure of a MnO_2_ and NiO containing electrode is not maintained during cycling.

## Graphical abstract

The air electrode is the very heart of a metal–air battery and needs to be highly efficient and stable. MnO_2_ is an efficient catalyst for the discharging reaction but is not stable during the charging of the battery. The investigation of the electrode composition toward the electrocatalytic activity and stability indicates that a partial substitution of MnO_2_ by NiO is highly beneficial for the cycling stability.

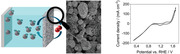

## 1 Introduction

Defined in the Paris Agreement under the United Nations Framework Convention on Climate Change, one important goal is to keep the increase of the global average temperature below 2 °C as compared to the pre‐industrial level.^[^ ^1^ ^]^ According to the United States Environmental Protection Agency (EPA), the electricity‐ and transportation‐sector combined were responsible for more than 50 % of the total greenhouse gas emissions in 2020.^[^ ^2^ ^]^ Thus, a large proportion of the consumption of electrical energy needs to be covered by renewable sources. However, the generation of electrical energy from renewable sources is subjected to natural fluctuations and needs to be stored, for example, as electrochemical energy in batteries.

In conventional batteries, energy is stored in two electrodes referred to as anode and cathode. The capacity of the battery is limited by the electrode with the lower capacity. In metal–air batteries (MABs), the conventional cathode is replaced by an “air‐breathing” electrode, meaning oxygen is reduced during discharging and evolved during charging of the battery. Herein, the active material at the cathode side of a MAB is supplied by ambient atmosphere and, consequently, does not need to be stored within the battery. Hence, MABs offer a higher specific capacity compared to other battery technologies.

One of the main challenges in constructing MABs, that can compete with established battery technologies, is the development of highly active and stable bifunctional air electrodes. Air electrodes that support either the oxygen reduction or evolution are already commercialized for fuel cells, primary MABs, and water‐electrolyzers. Within secondary MABs, oxygen‐reduction and‐evolution can either be decoupled, i.e., take place at two separated electrodes,^[^ ^3^ ^]^ or take place at one bifunctional air electrode. The latter has several advantages concerning weight, cell size, material costs, and convenience during operation. To construct a bifunctional air electrode, either a bifunctional catalyst or a mixture of an oxygen reduction reaction catalyst (ORR) and an oxygen evolution reaction (OER) catalyst is used to reduce the overpotentials of the corresponding oxygen reactions. If a mixture of an ORR and OER catalyst is used, both reactions can also be decoupled by designing a bifunctional air electrode that consists of two catalyst layers.^[^ ^3^, ^4^, ^5^, ^6^ ^]^

Along this line, MnO_2_ is an established catalyst for the ORR, commonly used in primary zinc‐air batteries and also being considered for bifunctional air electrodes due to its abundance and low cost. Even though MnO_2_ is a superior catalyst for the ORR, its OER activity is low (882 mV versus Hg/HgO @ 10 mA cm^−2^) compared to other catalysts that are active for the OER, such as La_0.6_Sr_0.4_CoO_3_ (760 mV vs Hg/HgO @ 10 mA cm^−2^), NiCo_2_O_4_ (716 mV vs Hg/HgO @ 10 mA cm^−2^), or NiFe‐LDH (569 mV vs Hg/HgO @ 10 mA cm^−2^).^[^ ^7^ ^]^ Manganese dioxide (MnO_2_) is a polymorph that occurs in several modifications.^[^ ^8^ ^]^ The ORR activity depends on the Mn‐modification according to: *β*‐MnO_2_ < *λ*‐MnO_2_ < *γ*‐MnO_2_ < *α*‐MnO_2_ ≈ *δ*‐MnO_2_.^[^ ^9^ ^]^ Previous approaches to increase the OER activity of MnO_2,_ e.g., included doping with Ru, Ir, Ni, Fe, V, and Co^[^ ^3^, ^4^, ^5^, ^6^, ^10^, ^11^, ^12^, ^13^, ^14^ ^]^ as well as sulfidation.^[^ ^6^ ^]^ Furthermore, its integration into a hybrid catalyst or mixing with an OER active catalyst is quite common in literature.^[^ ^4^, ^15^, ^16^, ^17^, ^18^, ^19^, ^20^, ^21^, ^22^, ^23^, ^24^, ^25^, ^26^, ^27^, ^28^, ^29^, ^30^, ^31^, ^32^, ^33^, ^34^ ^]^

Since the electronic conductivity of metal‐oxide catalysts is low compared to metallic catalysts, they are typically mixed with conductive additives and major work on MnO_2_‐based catalysts bifunctional air electrodes has been performed in carbon‐based systems.^[^ ^4^, ^12^, ^15^, ^16^, ^17^, ^22^, ^24^, ^25^, ^28^, ^29^, ^30^, ^35^, ^36^, ^37^, ^38^, ^39^, ^40^, ^41^ ^]^ However, the long‐term stability of carbon‐based bifunctional air electrodes is not satisfactory due to the carbon corrosion potential being close to that of the OER.

Contrary, only minor work has been devoted to bifunctional air electrodes with a carbon‐free conductive additive in the catalyst layer. Herein, nickel is a common substitute for carbon^[^ ^5^, ^6^, ^29^, ^40^, ^42^, ^43^ ^]^ and manganese‐oxide catalyzed, nickel‐based bifunctional air electrodes were reported to be stable in nickel‐based electrodes under mild conditions (≤0.1 m KOH and ≤10 mA cm^−2^)^[^ ^42^ ^]^ but not under harsher and industrially more relevant environments (≥6 m KOH and ≥10 mA cm^−2^).^[^ ^6^, ^43^ ^]^ Stability of those electrodes was improved by physically decoupling ORR and OER using a catalyst mixture of MnO_2_ for the ORR and NiFe LDH for the OER in a Janus‐type electrode.^[^ ^5^ ^]^

A more practical solution to assemble bifunctional systems is to mix an ORR and OER catalyst within one active layer. Along this line, a carbon‐based electrode using MnO_2_ for ORR and NiCo_2_O_4_ for OER was reported by Flegler et al.^[^ ^4^ ^]^ The authors investigated three different bifunctional air electrodes: one with a MnO_2_ catalyst, one with a NiCo_2_O_4_ catalyst, and one with a mixture of MnO_2_ and NiCo_2_O_4_. The electrode with the mixed catalyst showed the highest stability. Marini et al. utilized a mixture of MnO_2_ and a Ni/NiO core/shell nanoparticles as a catalyst in a carbon‐based air electrode and reached a stable cycling behavior at a current density of 10 mA cm^−2^ for 100 cycles (400 h) in 6 m KOH.^[^ ^29^ ^]^ Further studies conducted by Marini et al. indicate that the addition of Ni/NiO nanoparticles reduces *α*‐MnO_2_ dissolution during OER.^[^ ^44^ ^]^

The results by Flegler et al. and Marini et al. indicate that mixing MnO_2_ with an additional OER active catalyst is beneficial for the long‐term stability. Thus, using mixed‐catalyst bifunctional air electrodes seems to be an attractive option toward the development of highly active and stable bifunctional air electrodes. However, the influence of a mixture of MnO_2_ with an additional OER catalyst in one catalyst layer on the stability of the bifunctional air electrode has not yet been investigated in a carbon‐free system.

To close this research gap, we herein studied the influence of a partial substitution of MnO_2_ by nickel‐oxide (NiO), which is a well‐known OER catalyst, on the electrocatalytic activity towards the oxygen reaction and the stability of carbon‐free bifunctional air electrodes. The herein developed bifunctional air electrode showed an initial activity of 0.77 V versus RHE @ ‐25 mA cm^−2^ (ORR) and 1.57 V vs RHE @ 25 mA cm^−2^ (OER). Furthermore, the potential increase after 60 cycles was 0.05 V for ORR and 0.04 V for OER at a cycling duration of 60 min (30 min ORR followed by 30 min OER). It can be shown that a partial substitution of MnO_2_ by NiO can drastically increase the long‐term stability of bifunctional air electrodes.

## 2 Results and Discussion

**Figure 1:**
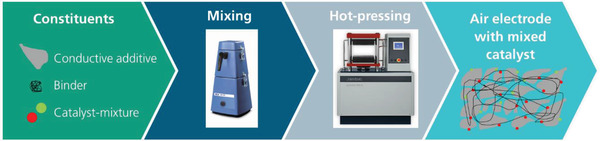
Flow‐chart to illustrate the manufacturing process of bifunctional air electrodes from a dry mixture via hot‐pressing, as applied in this work. The process consists of a total of three steps. The constituents of the electrode are mixed in a knife‐mill and subsequently pressed on a nickel‐mesh under elevated temperature.

Bifunctional air electrodes were prepared from a dry blend of nickel (used as conductive additive), PTFE (used as binder), and a catalyst mixture (MnO_2_ and NiO in a ratio of 50:50 by weight). The components were mixed in a knife mill to obtain a homogenous blend that was subsequently hot‐pressed on a nickel screen to obtain the final electrode. The process is illustrated in the flowchart as shown in **Figure** **1**.

**Figure 2:**
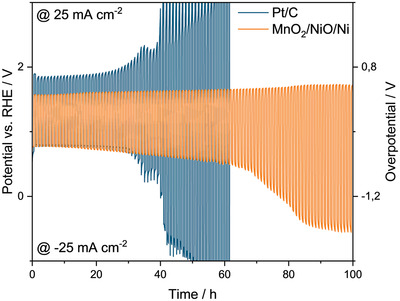
CP measurements with ‐25 mA cm^2^ and 25 mA cm^2^ for ORR and OER, respectively, applied for 30 minutes each to the MnO_2_/NiO/Ni bifunctional air electrode and commercial Pt/C electrode for reference.

To judge whether this electrode system is suited as a bifunctional air electrode for metal–air batteries, we first compared the activity and stability of the bifunctional electrode with a commercially available Pt/C air electrode. **Figure** **2** shows a chronopotentiometric (CP) measurement of the MnO_2_/NiO/Ni bifunctional air electrode as well as the commercial Pt/C electrode over 100 h at the same experimental conditions. CP measurements of the MnO_2_/Ni and NiO/Ni electrodes are provided in Figure S1 (Supporting Information). Notably, the bifunctional air electrode prepared within this work shows a similar initial ORR overpotential and a lower initial OER potential than the Pt/C reference electrode. Furthermore, the bifunctional air electrode shows no significant potential increase within the first 60 h of the CP measurement. In contrast, the overpotentials for ORR and OER of the Pt/C electrode continuously increased from cycle 30 on.

**Table** **1** shows the characteristics of bifunctional air electrodes from other works compared to the herein developed MnO_2_/NiO/Ni bifunctional air electrode. The initial activity (0.77 V vs RHE @ ‐25 mA cm^−2^ and 1.57 V vs RHE @ 25 mA cm^−2^) of the MnO_2_/NiO/Ni bifunctional air electrode, developed in this work, is comparable to silver‐based bifunctional air electrodes and superior to most literature reported noble metal and carbon‐free electrodes.

**Table 1:** Characteristic data of reported bifunctional air electrodes with a carbon‐free conductive additive compared with the data from this work. For convenience, all potentials are converted to RHE. The used formulas are provided in Equations S1 to S4 (Supporting Information)

Composition catalyst layer	Manufacturing method	Performance ORR	Performance OER	Measurement Setup	Conditions	Refs.
Nickel particles MnO_2_ NiO	Hot‐pressing	‐25 mA cm^−2^ @ 0.77 V	25 mA cm^−2^ @ 1.57 V	Half cell vs RHE	6 m KOH RT Synthetic air	This work
Nickel particles Silver	Sintering	‐150 mA cm^.2^ @ 0,62 V	Not studied	Half cell vs Hg/HgO	5 m KOH 40 °C 20 mbar	[45]
Nickel foam NiCo_2_O_4_	Pressing with subsequent thermal treatment	−20 mA cm^−2^ @ 0.82 V −100 mA cm^−2^ @ 0.62 V	20 mA cm^−2^ @ 1.52 V 100 mA cm^−2^ @ 1.62 V	Half cell vs Hg/HgO	8 m NaOH 60 °C Oxygen 200 mL min^−1^	[46]
Silver particles Co_3_O_4_	Pressing with subsequent thermal treatment	−120.5 mA cm^−2^ @ 0.3 V	54.3 mA cm^−2^ @ 1.8 V	Half cell vs RHE	1 m LiOH 25 °C Oxygen	[47]
Silver particles IrO_2_@TiO_2_ ^a)^	Pressing with subsequent thermal treatment	−116 mA cm^−2^ @ 0,3 V	66,6 mA cm^−2^ @ 1.8 V	Half cell vs RHE	1 m LiOH 25 °C Oxygen	[47]
Nickel particles Co_3_O_4_	Pressing with subsequent thermal treatment	−35 mA cm^−2^ @ 0.3 V	45 mA cm^−2^ @ 1.8 V	Half cell vs RHE	1 m LiOH 25 °C Oxygen	[48]
Nickel foam MnO_2_ CNTs	Hydrothermal method and subsequent thermal treatment	−20 mA cm^−2^ @ 1.16 V −50 mA cm^−2^ @ 0.98 V	20 mA cm^−2^ @ 1.99 V 50 mA cm^−2^ @ 2.1 V	Full cell vs Zn/Zn^2^	6 m KOH with 0.4 m ZnO Ambient air	[40]
Nickel foam MnO_2_ NiFe	Galvanic deposition	−40 mA cm^−2^ @ 0.4 V	80 mA cm^−2^ @ 1.7 V	Half cell vs Hg/HgO	1 m KOH O_2_ saturated	[5]
Nickel foam MnO_2_ NiFe	Galvanic deposition	−50 mA cm^−2^ @ 1.16 V	50 mA cm^−2^ @ 2.16 V	Full cell vs Zn/Zn^2+^	6 m KOH with 0.2 m Zn(CH_3_COO)_2_	[5]
Nickel foam MnO_2_	Hydrothermal method	−20 mA cm^−2^ @ 1.06 V	20 mA cm^−2^ @ 2.31 V	Full cell vs Zn/Zn^2+^	7 m KOH with 4 wt% ZnO 30°C Ambient atmosphere	[6]
Nickel foam Co_3_O_4_	Hydrothermal method	−10 mA cm^−2^ @ 1.21 V	10 mA cm^−2^ @ 2.16 V	Full cell vs Zn/Zn^2+^	6 m KOH with 0.2 m Zn acetate	[49]

^a)^ IrO_2_ coated TiO_2_.

To evaluate whether the substitution of carbon by nickel can increase the stability of bifunctional air electrodes with multiple catalysts, we compared the performance decay of the herein investigated electrode to examples from literature (see **Table** **2**).

**Table 2:** Stabilities of carbon‐based bifunctional air‐electrodes from literature. The stability can be defined as the potential change at a constant current within a certain number of cycles

Composition catalyst layer	Manufacturing method	Performance decrease	Cycling protocol	Conditions	Refs.
MnO_2_ Ni (mixture)	Hot‐pressing	Potential increase of 57% for ORR and 6% for OER	25 mA cm^−2^ 30 min	6 m KOH RT Synthetic air	This work
Co_3_O_4_ modified MnO_2_ nanotubes (hybrid)	Spraying	Potential increase of 5% after 60 cycles	15 mA cm^−2^ 7 min	6 m KOH 25 °C	[15]
MnO_2_ NiCo_2_O_4_ (mixture)	Screen‐printing	Stable for 50 cycles	2.5 mA cm^−2^ 200 s	6 m KOH RT Air	[4]
La_2_O_3_ Co_3_O_4_ MnO_2_‐CNTs (hybrid)	Spraying	Stable for 21 cycles	50 mA cm^−2^ 30 min	6 m KOH 25°C	[22]
NiCo_2_O_4_ MnO_2_‐CNTs (hybrid)	Spraying	Increase voltage gap from 0.77 V vs Zn/Zn^2+^ to 1.5 V vs Zn/Zn^2+^ after 4560 cycles	20 mA cm^−2^ 5 min	6 m KOH	[25]
MnO_2_ Ni/NiO (mixture)	Spraying	Stable for 100 cylces	10 mA cm^−2^ 2 h	6 m KOH 21°C	[29]
MnO_2_ NiFe‐LDHs (hybrid)	Roll pressing	1.15 V vs Zn/Zn^2+^ to 1.08 V vs Zn/Zn^2+^ for ORR and 2.25 V vs Zn/Zn^2+^ to 2.08 V vs Zn/Zn^2+^ for OER after 20 cycles	25 mA cm^−2^ 1 h	33% KOH	[30]

We observed an initial ORR potential of about 0.77 V versus RHE and an initial OER potential of about 1.57 V versus RHE. After 60 cycles the ORR potential was 0.49 V versus RHE and the corresponding OER potential 1.66 V versus RHE. This corresponds to an increase of the ORR overpotential from cycle one to 60 of about 57% and to an increase of the OER overpotential of about 6 %. Furthermore, the electrode investigated in our work showed an initial potential gap of 0.8 V. The potential gap increased to 1.17 V after 60 cycles and to 2.28 V after 100 cycles (1 cycle corresponds to 1 h).

A direct comparison of the cycling stabilities to other works is not trivial due to the deviating conditions and cycling protocols used in different works. However, one can estimate some trends. We found two investigations of a mixed catalyst within a carbon‐based bifunctional air electrode. Flegler et al. and Marini et al. used a comparable low cycling current densities of 2.5 mA cm^−2^ to 10 mA cm^−2^.^[^ ^4^, ^29^ ^]^ Higher current densities were utilized by Xu et al.^[^ ^22^ ^]^ The authors presented a carbon‐based air electrode with a ternary hybrid‐catalyst composed from La_2_O_3_/Co_3_O_4_/MnO_2_‐CNTs showed no potential increase after 21 h at a 50 mA cm^−2^ load (1 cycle corresponds to 1 h). The herein investigated bifunctional air electrode showed a potential of 0.72 V versus RHE and 1.61 V versus RHE at cycle 21 for ORR and OER, respectively. This corresponds to a voltage increase of 0.05 V for ORR and 0.04 V for OER. However, a hybrid catalyst that includes CNTs was used in this publication. Despite the high cycling stability of this electrode, one needs to consider that synthesizing hybrid catalysts is elaborate and needs multiple steps that might not be suited for industrial applications.

The performance after 60 cycles of the herein developed electrode requires further improvement. However, this electrode system is an attractive alternative to other approaches due to its high initial activity (ORR potential of 0.77 V versus RHE and OER of 1.57 V versus RHE), absence of noble‐metals, cobalt, and rare‐earth elements, exclusion of carbon corrosion, and simple manufacturing process. To investigate the reasons for the potential increase and, thus, electrode failure, further investigations were conducted.

### 2.1 From Rotating Disk to Gas Diffusion Electrodes

**Figure 3:**
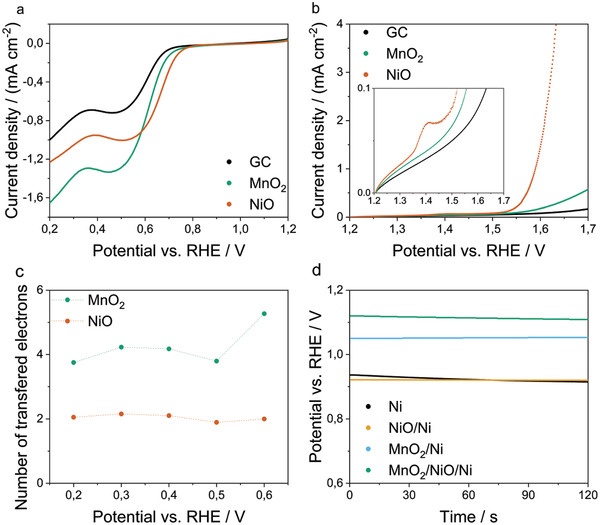
LSV curves of the catalyst measured on RDE setup, obtained at a rotation rate of 900 rpm for a) ORR and b) OER. The curve of the measurement of the plain substrate is denoted as GC for glassy carbon. The inlet shows the region at low current densities. c) Electron‐transfer‐number at different potentials obtained from a linear regression of the corresponding KL‐plots. d) Open circuit potential as a function of time of bifunctional air electrodes with all tested catalyst combinations.

The open‐circuit potential (OCP) is a crucial parameter for a MAB since the energy density is proportional to the OCP and directly linked to the ORR reaction mechanism at the catalyst. Hence, regarding the efficiency of MABs, only catalysts that catalyze the direct oxygen reduction should be considered for incorporation into GDEs for MABs. A fast and reliable method to determine the reaction mechanism on a catalyst's surface is the Koutecky‐Levich (KL) analysis of LSVs from RDE measurements, obtained at variating rotations rates. From **Figure** **3**a follows that both catalyst samples show higher limiting current densities than the glassy carbon (GC) reference. While the GC reference shows a current density of 1 mA cm^−2^, MnO_2_ shows a current density of 1.65 mA cm^−2^ and NiO shows a current density of 1.23 mA cm^−2^ for ORR at 0.2 V versus RHE. Comparing the MnO_2_ and NiO catalysts, the overpotential of the NiO catalyst is lower compared to MnO_2_ up to a current density of about 1 mA cm^−2^. This behavior reverses for current densities higher than 1 mA cm^−2^.

Figure 3b shows the linear sweep voltammograms (LSV) of the OER. The exposed peak at about 1.4 V of the NiO sample can be related to the reaction of Ni(OH)_2_ to NiO(OH)^[^ ^50^, ^51^ ^]^ and the highest activity towards OER was found at a current density of 1.46 mA cm^−2^ compared to MnO_2_ with a current density of 0.18 mA cm^−2^ at 1.6 V versus RHE and GC (0.08 mA cm^−2^ @1.6 V versus RHE). The resulting electron transfer numbers for the ORR from the slope of the KL plot (Figure S2, Supporting Information) at chosen potentials are shown in Figure 3c (calculation details are provided in the Supporting Information). Herein, NiO shows a constant electron transfer number of two at all considered potentials. Contrary, MnO_2_ shows an electron transfer number of four up to 0.5 V versus RHE. The increase of the transferred electrons after 0.6 V versus RHE can be explained by the higher offset potential of the ORR at the MnO_2_ catalyst. Importantly, the KL equation is only valid in the diffusion‐controlled regime, thus, calculations were performed from 0.2 V versus RHE to 0.6 V versus RHE. Based on these results, the tested MnO_2_ catalyst catalyzes the oxygen reduction to water and the tested NiO catalyst catalyzes the oxygen reduction to hydrogen peroxide within the considered potential range.

For further studies, the catalysts (NiO/Ni and MnO_2_/Ni) and a mixture of both catalysts (MnO_2_/NiO/Ni) were implemented into gas diffusion electrodes that were prepared according to the procedure that is shown in Figure 1. An additional reference sample that contains no additional catalyst (Ni) was also prepared to study the intrinsic catalytical properties of the nickel powder, used as conductive additive.

Figure 3d shows the measured OCP as a function of time over a period of 120 s. All electrodes show a stable OCP over this period indicating that no corrosion takes place at the electrode/electrolyte interface when no current is transferred through the cell. The electron transfer number of the catalyst directly influences the OCP of the bifunctional gas diffusion electrode and all electrodes that include MnO_2_ show a higher OCP than all other samples. This indicates that the direct mechanism governs the ORR at the MnO_2_ containing electrodes and the indirect mechanism at the NiO/Ni and Ni bifunctional air electrodes.

**Figure 4:**
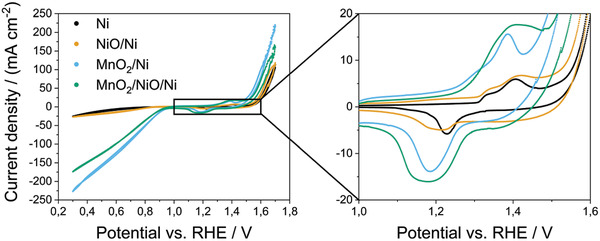
Cyclic voltammograms (second cycle) of bifunctional air electrodes with all tested catalyst combinations. The enlarged section highlights the corrosion reactions of the electrode materials. The region is indicated by a rectangle.

The cathodic currents of the cyclic voltammograms of the bifunctional air electrodes with the corresponding catalysts in **Figure** **4** correspond to reductions and anodic currents to oxidation reactions. Seemingly, no diffusion limitation occurs within the regimes of the oxygen reactions because the current continuously increases proportionally to the applied potential.

Contrary to RDE measurements, the MnO_2_/Ni bifunctional air electrode shows the highest activity towards ORR and OER within the considered potential range. The GDE setup is far more complex: Among factors, such as conductivity and hydrophobicity, the pore structure of the bifunctional air electrode is crucial for the performance. NiO showed the highest electrocatalytic activity towards OER when tested in RDE measurements. Yet, it needs to be considered that these RDE experiments, with a controlled diffusion of the reactants to the active sites of the electrode, deviate from the environment of the catalyst operated in real working conditions, such as the GDE setup.^[^ ^52^ ^]^

Concerning the results of the GDE measurements, shown in Figure 4, the cyclic voltammograms of the NiO/Ni and Ni samples do not show any significant difference. Either the NiO catalyst is inactive in the bifunctional air electrode or the OER current can also be limited by the ability of the bifunctional air electrode to release oxygen. Oxygen bubbles might block the active sites of the electrode of these samples at high current densities. This is indicated by the highly fluctuating currents at high anodic potentials. As can be seen in Figure 4, the OER activity of NiO/Ni is slightly higher than the activity of Ni at low current densities (overpotentials of 315 mV for NiO/Ni and 337 mV for Ni at 10 mA cm^−2^).

The Ni bifunctional air electrode shows an anodic peak at 1.41 V versus RHE and a cathodic peak at 1.23 V versus RHE. The NiO/Ni bifunctional air electrode shows an anodic peak at 1.42 V versus RHE and a cathodic peak at 1.21 V versus RHE. The MnO_2_/Ni bifunctional air electrode shows an anodic peak at 1.39 V versus RHE and a cathodic peak at 1.19 V versus RHE. The MnO_2_/NiO/Ni bifunctional air electrode shows an anodic peak at 1.41 V versus RHE and a cathodic peak at 1.18 V versus RHE. The anodic peaks feature a shoulder and are attributable to Ni(II) to Ni(III) oxidation while the cathodic peaks are attributable to Ni(III) to Ni(II) reduction. Thus, the Ni system is prone to corrosion that is partially reversible or reversible. This can be concluded from the presence of a pair of anodic peaks and cathodic peaks in the considered potential range.

Two potential reactions could explain the anodic peaks in Figure 4: The anodic peak at lower potentials corresponds to the *β*‐Ni(OH)_2_ to *β*‐Ni(OOH) oxidation and the anodic peak at higher potentials to the *α*‐Ni(OH)_2_ to *γ*‐Ni(OOH) oxidation.^[^ ^53^ ^]^ Alternatively, the shoulder could also correspond to the *β*‐Ni(OH)_2_ to *β*‐Ni(OOH) and the anodic peak to the *β*‐Ni(OOH) to *γ*‐Ni(OOH) transition.^[^ ^54^ ^]^ Ni(OH)_2_ is initially formed on nickel metal when exposed to air^[^ ^55^ ^]^ or being submerged in an aqueous solution.^[^ ^56^ ^]^ A combination of both mechanisms is also possible. Oxidations of nickel species take place at potentials around 1.38 V to 1.58 V versus RHE^[^ ^57^, ^58^ ^]^ that can also overlay with the OER. In contrast to the anodic branch of the CV, only one cathodic peak is visible in the CVs shown in Figure 4. This behavior indicates that one of the anodic reactions is not reversible or the peaks are overlaying. This peak is mainly associated with the *β*‐Ni(OOH) to Ni(OH)_2_ reduction.^[^ ^50^, ^59^ ^]^

### 2.2 Stability

**Figure 5:**
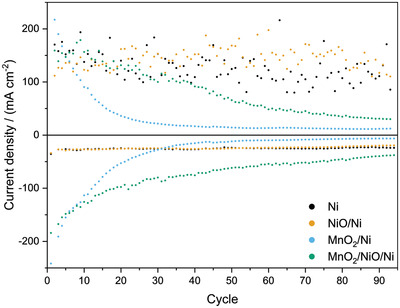
Current densities at 0.3 and 1.7 V for ORR and OER, respectively, obtained from 100 CV cycles of the bifunctional air electrode without catalyst and all catalyst combinations (e). Positive current densities are associated with OER and negative current densities with ORR.

As shown in **Figure** **5**, the Ni and NiO/Ni bifunctional air electrodes show constant current densities for ORR and OER. The current densities of the MnO_2_‐containing bifunctional air electrodes continuously decrease. It is also noticeable that the current densities of the MnO_2_/NiO/Ni bifunctional air electrode decrease slower than the current densities of the MnO_2_/Ni bifunctional air electrode with increasing cycling numbers. The MnO_2_/Ni electrode shows an initial current density of 242 mA cm^−2^ and 217 mA cm^−2^ for ORR and OER, respectively. The corresponding currents decreased to 15 mA cm^−2^ and 17 mA cm^−2^ when reaching cycle 40. Compared to this sample, the initial current densities of the MnO_2_/NiO/Ni electrode are 184 mA cm^−2^ for the ORR and 159 mA cm^−2^ for the OER. This sample still shows a current density of 76 mA cm^−2^ for the ORR and 92 mA cm^−2^ for the OER at cycle 40. This decrease corresponds to a current decrease from cycle 1 to 40 of 94% for the ORR and 92% for the OER for the MnO_2_/Ni electrode compared to an ORR decrease of 59% and an OER current decrease of 43% for the MnO_2_/NiO/Ni electrode. The higher current densities at higher cycles of the NiO‐containing sample, compared to the NiO‐free sample, conclude that a partial substitution of MnO_2_ by NiO increases the stability of the electrode. Even though the Ni and NiO/Ni bifunctional air electrodes are stable, the initial current density of the Ni electrode is just 36 mA cm^−2^ for the ORR and 170 mA cm^−2^ for the OER. The initial ORR current density of the NiO/Ni electrode is 34 mA cm^−2^ and the initial OER current density of the NiO/Ni‐electrode is 112 mA cm^−2^. All four values are significantly lower than the current densities observed for electrodes that contain MnO_2_.

In contrast to the RDE results, the electrodes that contain MnO_2_ also show higher OER current densities (217 mA cm^−2^ for MnO_2_/Ni and 159 mA cm^−2^ for MnO_2_/NiO/Ni) than the Ni (170 mA cm^−2^) and the NiO/Ni electrode (112 mA cm^−2^).

**Figure 6:**
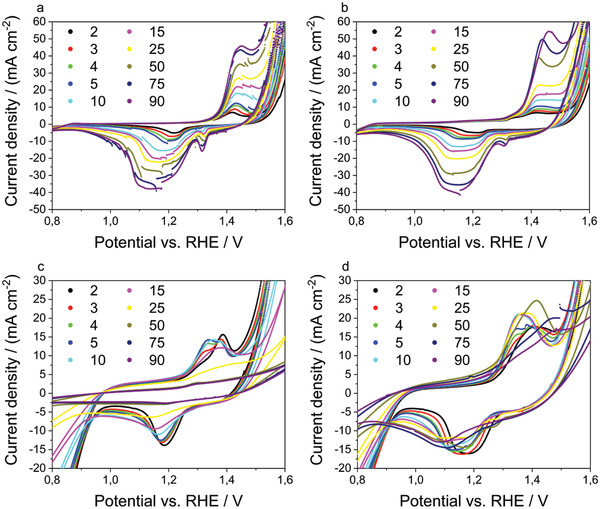
Enlarged section of cyclic‐voltammograms of the a) Ni, b) NiO/Ni, c) MnO_2_/Ni, and d) MnO_2_/NiO/Ni bifunctional air electrodes. The corresponding cycle numbers are provided in the legend. The plots are enlarged to highlight the potential region in which the corrosion reactions of the electrode are visible.

The sudden current density changes of the CVs, shown in **Figure** **6**a,b, stem from remaining oxygen bubbles on the electrode surface. Furthermore, the anodic shoulder vanishes after a few cycles and a second cathodic peak forms at about 1.3 V versus RHE. This peak can be associated with the transition of *γ*‐Ni(OOH) to *α*‐Ni(OH)_2_ and is an indicator for a growing Ni‐(oxy)hydroxide layer on the nickel particles or the NiO particles.^[^ ^53^ ^]^ This assumption is supported by the increase of the peak intensities of the Ni bifunctional air electrode (Figure 6a) and NiO/Ni bifunctional air electrode (Figure 6b) with an increasing number of cycles.^[^ ^53^ ^]^

**Figure 7. a):**
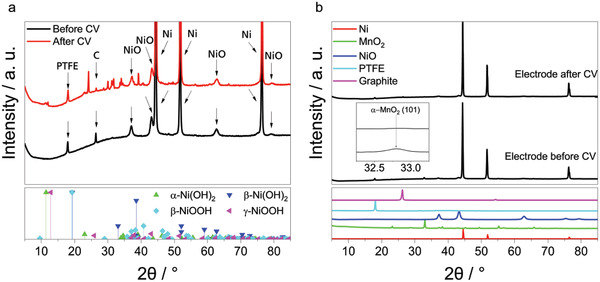
XRD spectra of the NiO/Ni bifunctional air electrode before and after CV measurements. The references of the corresponding nickel (oxide) hydroxides are shown at the bottom of the plot. XRD spectra of MnO_2_/NiO/Ni bifunctional air electrode before and after cycling b) and the corresponding constituents (colored lines in the lower frame) of the electrode. Enlarged part of the spectrum with the corresponding reflex of the (101) plane of *α*‐MnO_2_ indicated. The peak that corresponds to graphite is attributed to remaining graphite of the plate that was placed on the electrode during hot pressing.

In literature, this behavior is explained by the growth of a porous nickel (oxy)hydroxide layer and a subsequent increase of interfacial area between electrode and electrolyte.^[^ ^53^, ^60^ ^]^ This observation is supported by the appearance of peaks that correspond to multiple Ni(OH)_2_ and NiOOH phases in the XRD spectrum in **Figure** **7**a. However, the corrosion behavior does not influence the cycling stability of this sample since the ORR and OER currents remain stable with increasing cycle number.

Concerning the MnO_2_/NiO/Ni sample, it is not trivial to assign all peaks to corresponding oxidations, reductions, and transitions since there are multiple species present in the bifunctional air electrode. The MnO_2_‐powder used in this work is commercially available (see Section 4.1 for details) and consists of multiple MnO_2_ modifications (the corresponding XRD‐spectrum is shown in Figure S3, Supporting Information). Furthermore, the MnO_2_ involving reactions occur simultaneously with the Ni‐based reactions. The MnO_2_/NiO/Ni bifunctional air electrode shows a similar electrochemical behavior as the MnO_2_/Ni bifunctional air electrode. The MnO_2_/NiO/Ni bifunctional air electrode shows the best compromise between activity and stability with an initial current density of 242 mA cm^−2^ for ORR, 217 mA cm^−2^ for OER and a decrease of the current densities from cycle one to 40 of 59% for ORR and 43% for OER.

**Figure 8:**
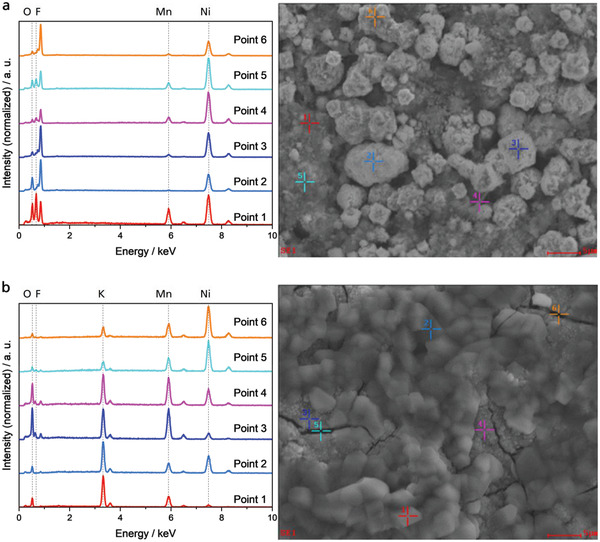
EDX spectra, performed at several points indicated within the SEM images of the MnO_2_/NiO/Ni bifunctional air electrode before a) and after b) cycling.

**Figure 9:**
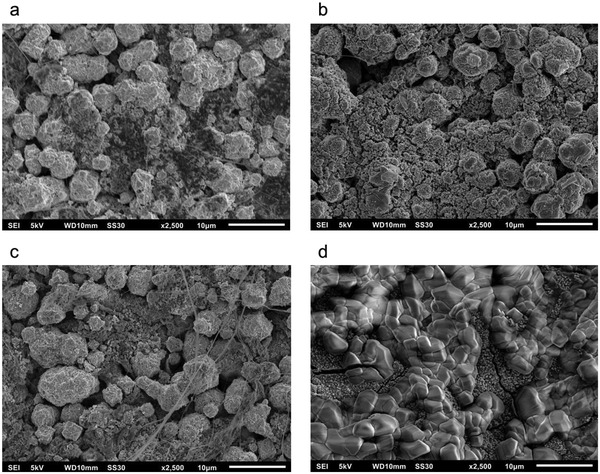
SEM images of the NiO/Ni and MnO_2_/NiO/Ni bifunctional air electrode before a,c) cycling and after b,d) cycling at a magnification of 2500.

Contrary to the behavior of the NiO/Ni and Ni bifunctional air electrodes, the intensities of the peaks, observed in the CVs of MnO_2_/Ni bifunctional air electrode, decrease with an increasing cycle number. Within the first cycles, the anodic peak at 1.39 V versus RHE decreases while the shoulder increases until two peaks of the same height are present. Subsequently, both peaks merge and become smaller with increasing cycles. In addition, the decrease of the CV peaks in Figure 6c,d occurs together with the decrease of the ORR and OER currents of these samples. This might be explained by a reduction of the electronic conductivity of the electrode due to the dissolution of MnO_2_ into the electrolyte which results into a disruption of conductive paths. The two XRD spectra shown in Figure 7b of the bifunctional air electrode before and after cycling are mostly redundant. An exception is the corresponding peak assignable to the (101)‐plane of *α*‐MnO_2_, which does not appear in the cycled sample and, thus, shows the structural integrity of most of the components of the bifunctional air electrode. Contrary, the MnO_2_ catalyst transforms into an amorphous phase or dissolves in the electrolyte. Since manganese was detected on the electrode surface after cycling via EDX (**Figure** **8**), formation of an amorphous phase seems to be a feasible pathway alike formation of a very thin layer of a crystalline phase that is beyond the sensitivity of the used XRD. This hypothesis is also supported by SEM images shown in **Figure** **9**. While the morphology of the NiO/Ni bifunctional air electrode after cycling (Figure 9b) is similar to the one before cycling (Figure 9a), the morphology of the bifunctional air electrode that contains MnO_2_ and NiO completely changed. The MnO_2_/NiO/Ni bifunctional air electrode has a porous structure before cycling (Figure 9). After cycling, a rough, uneven structure is visible. The electrode also shows some arbitrarily oriented cracks (Figure 9d). These cracks are typical for cycled electrodes and can be attributed to washing and drying.^[^ ^61^ ^]^ XRD and SEM/EDX data of the MnO_2_/Ni and Ni bifunctional air electrodes can be found in Figures S4 to S6 (Supporting Information).

From XRD and EDX data as well as SEM micrographs after cycling, we conclude that the MnO_2_/NiO/Ni bifunctional electrode was passivated during cycling due to the dissolution of MnO_2_ and redistribution on the surface of the bifunctional air electrode.

This study shows that the presented electrode composition shows a promising initial activity, includes no noble metals, and is easy to fabricate. Although the cycling stability is not satisfactory, we were able to show that the cycling stability can be improved by a partial substitution of MnO_2_ by NiO. From these findings, one might suggest to further optimizing the MnO_2_/NiO ratio with respect to the cycling stability of the electrode.

## 3 Conclusion

Within this work, we presented a highly active carbon‐, cobalt‐, and noble‐metal‐free MnO_2_/NiO‐based bifunctional air electrode for metal–air batteries in alkaline electrolyte. The bifunctional air electrodes were prepared by hot pressing and their activity towards ORR and OER was investigated via CV and CP measurements. The bifunctional air electrode that contains nickel as conductive additive and MnO_2_ as a catalyst (denoted as MnO_2_/Ni) reached an initial ORR current density of 242 mA cm^−2^ and an initial OER current density of 217 mA cm^−2^_._ Substituting the MnO_2_ catalyst by a catalyst mixture of MnO_2_ and NiO (denoted as MnO_2_/NiO/Ni) led to a decrease of the initial current density to 184 mA cm^−2^ and 159 mA cm^−2^ for the ORR and OER, respectively. XRD and SEM/EDX measurements were performed before and after cycling to investigate structural changes of the bifunctional air electrodes. Notably, only the bifunctional air electrodes that did not contain any MnO_2_ showed stable current densities over 100 CV cycles. XRD measurements indicate that a nickel (oxy)hydroxide layer grew on the electrode surface during cycling. Despite this, samples that contained MnO_2_ showed a superior initial activity and an elevated OCP due to the direct ORR mechanism that is catalyzed by MnO_2_. Noteworthy, the partial substitution of MnO_2_ by NiO drastically increased the cycling stability of the electrode from a relative current decrease of 94 % to 59 % for the ORR and from 92 % to 43 % for the OER within the first 40 cycles of a cycling test conducted by means of CV. Transformation of MnO_2_ into an amorphous phase as well as a surface restructuring was observed within the MnO_2_ and NiO‐containing sample. In summary, we show that bifunctional air electrodes with a MnO_2_ catalyst are highly active but reveal limited cycling stability. These bifunctional air electrodes are furthermore well‐suited for short‐term applications as electrodes in secondary metal‐air batteries and other electrochemical reactors.

## 4 Experimental Section

### Preparation of Bifunctional Air Electrodes

Bifunctional air electrodes with mixed catalysts (noted as MnO_2_/NiO) consist of MnO_2_/NiO‐mixture (MnO_2_: US Research Nanomaterials Inc., 98%, 50 nm; NiO: IOLITEC, 99.5%, 20–30 nm; weight ratio 50:50) as a bifunctional catalyst, nickel powder as a conductive additive (Alfa Aesar, 99.9 %, APS 3–7 µm), and PTFE (3 m TF2029z) as a hydrophobic binder. The total composition by weight was 10% catalyst; 10 % PTFE and 80% nickel). The constituents were first mixed in a knife mill (IKA M20 with a star‐shaped cutter) for a total time of 1 min. Mixing was done within four intervals à 15 s with a 120 s break in between to allow the mixture to cool down. The knife mill was loaded with 90 g material for each run. A stainless‐steel frame (dimensions: 90 × 70 × 1 mm^3^) was placed on a nickel‐mesh (100 × 90 mm^2^ Haver & Boecker, mesh opening 0.45 mm, wire diameter 0.18 mm). 30 g of the mixture was filled in the stainless‐steel frame. Excess material was removed and the remaining material was distributed homogeneously within the frame. The frame was removed and an expanded graphite plate (SIGRAFLEX® L10010TH, 1 mm, SGL CARBON GmbH) was placed on the green body to avoid sticking of the metal plates of the press to the sample after hot pressing. If not otherwise specified, the bifunctional air electrodes were pressed with a Servitec Polystat 300s laboratory press at 260 °C and 20 MPa for ten minutes. Afterward the expanded graphite plate was removed and the bifunctional air electrodes were allowed to cool down at room temperature. The bifunctional air electrodes are denoted as Ni, MnO_2_/Ni, NiO/Ni, and MnO_2_/NiO/Ni.

### Material Analysis: XRD Analysis

XRD analysis was performed to investigate the structural stability of the bifunctional air electrodes during cycling. XRD analysis was done with an X'Pert Powder by PANalytical (PIXcel1D detector with 256 channels) using a copper source (acceleration voltage 45 kV; current 40 mA) in an angular range of 5° to 85°. A step size of 0.05° at a scanning speed of 0.027° s^−1^ was used. The reference spectra were calculated from CIF files that were obtained from the crystallographic open database (COD). The powder spectra were calculated via Bragg's equation using “VESTA” with a wavelength of 1.54059 Å. The COD indices are provided in **Table** **3**.

**Table 3:** COD references to calculate the theoretical XRD spectra

Structure	COD reference
*α*‐Ni(OH)_2_	9012316
*β*‐Ni(OH)_2_	1548811
*β*‐NiOOH	4109372
*γ*‐NiOOH	9012318

### SEM/EDX Analysis

SEM images were obtained to study the morphology before and after cycling. A Jeol JSM‐6510 SEM and an acceleration voltage of 5 kV, a spot size of 30, and the secondary electron detector were used for all images. A detector by remX GmbH was used for the EDX measurements. To obtain the spectra, an acceleration voltage of 20 kV and a spot size of 60 were used. The K_*α*_‐lines are used to indicate the elements. MnO_2_ is represented by Mn and PTFE by F. The peaks that are not indicated correspond to the K_*β*_‐ or L_*α*_‐lines.

### Electrochemical Analysis: RDE Measurements

RDE studies were conducted to investigate the activity of the catalyst powders towards the oxygen‐reduction and ‐evolution reactions. In addition to that, Koutecky‐Levich analysis was performed to identify the oxygen‐reduction reaction mechanism. The measurements were conducted using a Metrohm Autolab rotator. A glassy carbon (GC) tip with a diameter of 3 mm was used for the experiments. Prior to each measurement, the tip was polished subsequently with 0.3 µm and 0.05 µm alumina suspension (MicroPolish Suspension, Buehler) for 10 min each on a synthetic rayon cloth pad (MicroCloth; Buehler) in an eight‐shaped pattern. The catalyst ink was prepared in a falcon tube by suspending 40 mg of the catalyst powder in a stock solution containing 4 mL 2‐propanol (Carl Roth; ROTISOLV ≥99,9%, UV/IR Grade), 80 µL Nafion solution (Alfa Aesar; D‐521 dispersion; 5 % w/w in water and 1‐propanol), and 15.92 mL deionized water (obtained from a Milli‐Q system; ionic resistance ≥ 18.2 MΩ). The ink was sonicated for 20 min in an ultrasonic bath and afterward mixed with a disperser (ULTRA‐TURRAX T18 digital; IKA) at 8000 rpm for 30 s. 3 µL of the catalyst ink was drop‐coated on the RDE tip before the tip was placed in an oven at 35 °C for about 20 min to allow the ink to dry. The measurements were done in 250 mL aqueous potassium hydroxide solution (Carl Roth; 1 m ± 0.2 %) in a 250 mL angled three neck round bottom flask. The electrolyte was purged with oxygen gas or nitrogen gas for oxygen‐reduction and ‐evolution measurements, respectively. The electrolyte was purged with about 100 mL min^−1^ during the measurements to ensure gas saturation of the electrolyte.

Activity measurements on the RDE were conducted by linear‐sweep‐voltammetry (LSV) in a potential range of 1.2 to 0.2 V versus RHE for ORR and 1.2 to 1.7 V versus RHE for OER using a Mini HydroFlex by Gaskatel as reference electrode, a platinum wire as a counter electrode, and a Gamry Interface 1010B potentiostat. Two LSVs were completed priory for conditioning purposes. Those were conducted at rotation rates of 100 rpm for ORR and 900 rpm for OER. ORR measurements were conducted using different rotation rates from 100 to 2500 rpm (100; 140; 250; 400; 900; 1600; 2500 rpm). OER measurements were conducted at a rotation rate of 900 rpm. Results are IR‐corrected by the integrated post‐run correction from the Gamry Echem Analyst software. The electrolyte resistance was determined prior to each measurement via impedance spectroscopy.

### Half‐Cell GDE Measurements

Electrochemical measurements of the bifunctional air electrodes were conducted in a half‐cell setup, using a commercial FlexCell PP by Gaskatel. The measurements were conducted at room temperature. 6 m aqueous potassium hydroxide (Merck EMSURE, ≥ 85%) solution was used as electrolyte. Synthetic air with a flow rate of about 20 mL min^−1^ was used as feeding gas. The cell pressure was controlled with a gas‐washing bottle (with a water level of about 2–3 cm).

A Gamry Interface 1010B potentiostat was used for all measurements. CV measurements were conducted between 0.3 V and 1.7 V versus RHE; starting and ending at 1.2 V versus RHE that is in about the range of the thermodynamic equilibrium potential of the ORR and OER. A Mini HydroFlex by Gaskatel was used as a reference electrode. A sweep rate of 1 mV s^−1^ was chosen for all CV measurements. CP measurements were conducted with an alternating current density of ‐25 mA cm^−2^ (ORR) and 25 mA cm^−2^ (OER) for 30 min each. Thus, 1 h corresponds to one cycle. The open‐circuit potential (OCP) was obtained prior to all CV and CP measurements.

## Conflict of Interest

The authors declare no conflict of interest.

## Supporting information

Supporting Information

## Data Availability

The data that support the findings of this study are available from the corresponding author upon reasonable request.

## References

[1] 7. d Paris Agreement. Paris, 12 December 2015, United Nations, Treaty Series 3156, https://treaties.un.org/Pages/ViewDetails.aspx?src=TREATY&mtdsg_no=XXVII‐7‐d&chapter=27&clang=_en (accessed: July 2022).

[2] https://www.epa.gov/ghgemissions/sources‐greenhouse‐gas‐emissions#:~:text=Transportation%20(27%25%20of%202020%20greenhouse,ships%2C%20trains%2C%20and%20planes (accessed: March 2023).

[3] P.‐C. Li , J. Power Sources 2016, 313, 37.

[4] A. Flegler , S. Hartmann , J. Settelein , K. Mandel , G. Sextl , Electrochim. Acta 2017, 258, 495.

[5] P. Wang , Y. Lin , L. Wan , B. Wang , ACS Appl. Mater. Interfaces 2019, 11, 37701. 31538761 10.1021/acsami.9b12232

[6] N. Radenahmad , R. Khezri , A. A. Mohamad , M. T. Nguyen , T. Yonezawa , A. Somwangthanaroj , S. Kheawhom , J. Alloys Compd. 2021, 883, 160935.

[7] A. Flegler , *PhD. Thesis*, Universität Würzburg, 2019.

[8] W. Wei , X. Cui , W. Chen , D. G. Ivey , Chem. Soc. Rev. 2011, 40, 1697. 21173973 10.1039/c0cs00127a

[9] Y. L. Cao , H. X. Yang , X. P. Ai , L. F. Xiao , J. Electroanal. Chem. 2003, 557, 127.

[10] A. J. Jeevagan , Y. Suzuki , T. Gunji , G. Saravanan , Y. Irii , T. Tsuda , T. Onobuchi , S. Kaneko , G. Kobayashi , F. Matsumoto , ECS Trans. 2014, 58, 9.

[11] J. Hao , Y. Liu , H. Shen , W. Li , J. Li , Y. Li , Q. Chen , J. Mater. Sci.: Mater. Electron. 2016, 27, 6598.

[12] Z. Ye , Adv. Funct. Mater. 2017, 27, 1704083.

[13] M. Lübke , ChemistrySelect 2018, 3, 2613.

[14] K. Selvakumar , ChemElectroChem 2022, 9, 202101303.

[15] G. Du , X. Liu , Y. Zong , T. S. A. Hor , A. Yu , Z. Liu , Nanoscale 2013, 5, 4657. 23608821 10.1039/c3nr00300k

[16] F. T. Goh , Z. Liu , X. Ge , Y. Zong , G. Du , T. A. Hor , Electrochim. Acta 2013, 114, 598.

[17] P. H. Benhangi , A. Alfantazi , E. Gyenge , Electrochim. Acta 2014, 123, 42.

[18] H. Jang , A. Zahoor , J. S. Jeon , P. Kim , Y. S. Lee , K. S. Nahm , J. Electrochem. Soc. 2015, 162, A300.

[19] K. Guo , Y. Li , J. Yang , Z. Zou , X. Xue , X. Li , H. Yang , J. Mater. Chem. A 2014, 2, 1509.

[20] J. Qiao , in ECS Meeting Abstracts, Vol. MA2016‐01, IOP Science, Bristol, UK 2016, p. 179.

[21] N. Xu , Y. Liu , X. Zhang , X. Li , A. Li , J. Qiao , J. Zhang , Sci. Rep. 2016, 6, 33590. 27646032 10.1038/srep33590 PMC5028838

[22] N. Xu , J. Qiao , X. Zhang , C. Ma , S. Jian , Y. Liu , P. Pei , Appl. Energy 2016, 175, 495.

[23] Y. J. Lee , D. H. Kim , T.‐G. Kang , Y. Ko , K. Kang , Y. J. Lee , Chem. Mater. 2017, 29, 10542.

[24] J. Wang , H. Li , N. Xu , J. Qiao , Ionics 2018, 24, 3877.

[25] N. Xu , Y. Cai , L. Peng , J. Qiao , Y.‐D. Wang , W. M. Chirdon , X.‐D. Zhou , Nanoscale 2018, 10, 13626. 29979460 10.1039/c8nr03162b

[26] M. F. Fink , ChemElectroChem 2020, 7, 4822.

[27] Y. Tang , H. Cheng , Q. He , H. Li , Y. An , J. Xie , R. Liu , J. Electrochem. Soc. 2020, 167, 160536.

[28] H.‐X. Huang , J.‐L. Liu , C. Wang , D.‐M. Liang , H.‐L. Wang , Catal. Lett. 2022, 152, 1040.

[29] E. Marini , L. Jörissen , S. Brimaud , J. Power Sources 2021, 482, 228900.

[30] X. Bao , K. Xie , Z. Zhang , Z. Liu , H. Zhou , F. Luo , D. Zhou , H.‐E. Wang , Ionics 2022, 28, 1273.

[31] N. Arshad , M. Usman , M. Adnan , M. T. Ahsan , M. R. Rehman , S. Javed , Z. Ali , M. A. Akram , G. P. Demopoulos , A. Mahmood , Nanomaterials 2022, 13, 99. 36616009 10.3390/nano13010099 PMC9823737

[32] M.‐R. Zamani‐Meymian , K. K. Chenab , H. Pourzolfaghar , ACS Appl. Mater. Interfaces 2022, 14, 55594. 36475585 10.1021/acsami.2c16826

[33] X. Zheng , A. M. Zuria , M. Mohamedi , Inorg. Chem. 2023, 62, 989. 36579965 10.1021/acs.inorgchem.2c03916

[34] T. Hu , W. Zhang , Z. Xia , Y. Zhu , Y. Liu , J. Zhang , L. Li , Int. J. Energy Res. 2022, 46, 11174.

[35] D. Kubo , K. Tadanaga , A. Hayashi , M. Tatsumisago , J. Mater. Chem. A 2013, 1, 6804.

[36] A. R. Mainar , L. C. Colmenares , O. Leonet , F. Alcaide , J. J. Iruin , S. Weinberger , V. Hacker , E. Iruin , I. Urdanpilleta , J. A. Blazquez , Electrochim. Acta 2016, 217, 80.

[37] K. Chen , M. Wang , G. Li , Q. He , J. Liu , F. Li , Materials 2018, 11, 601.29652850

[38] X. Li , F. Dong , N. Xu , T. Zhang , K. Li , J. Qiao , ACS Appl. Mater. Interfaces 2018, 10, 15591. 29616793 10.1021/acsami.7b18684

[39] X. Zheng , N. Mohammadi , A. Moreno Zuria , M. Mohamedi , ACS Appl. Mater. Interfaces 2021, 13, 61374. 34927435 10.1021/acsami.1c22371

[40] K. Xu , A. Loh , B. Wang , X. Li , J. Electrochem. Soc. 2018, 165, A809.

[41] N. Xu , J. Liu , J. Qiao , H. Huang , X.‐D. Zhou , J. Power Sources 2020, 455, 227992.

[42] A. Bekisch , K. Skadell , D. Poppitz , M. Schulz , R. Weidl , M. Stelter , J Endocr. Soc. 2020, 167, 144502.

[43] K. Xu , J. Song , P. Song , G. Xu , Z. Deng , J. Electrochem. Soc. 2020, 167, 130501.

[44] E. Marini , M. Liebert , F. Rossi , D. O. de Souza , P. Baumli , G. Aquilanti , F. Regnet , I. Lüdeking , B. Bozzini , L. Jörissen , S. Brimaud , J. Power Sources 2022, 546, 231879.

[45] L. Carlsson , L. Öjefors , J. Electrochem. Soc. 1980, 127, 525.

[46] X. Li , D. Pletcher , A. E. Russell , F. C. Walsh , R. G. Wills , S. F. Gorman , S. W. Price , S. J. Thompson , Electrochem. Commun. 2013, 34, 228.

[47] D. Wittmaier , N. Wagner , K. A. Friedrich , H. M. Amin , H. Baltruschat , J. Power Sources 2014, 265, 299.

[48] D. Wittmaier , S. Aisenbrey , N. Wagner , K. A. Friedrich , Electrochim. Acta 2014, 149, 355.

[49] W. Shang , W. Yu , Y. Ma , Y. He , Z. Zhao , M. Ni , H. Zhao , P. Tan , Adv. Mater. Interfaces 2021, 8, 2101256.

[50] D. S. Hall , C. Bock , B. R. MacDougall , J. Electrochem. Soc. 2013, 160, F235.

[51] J. Rozsypal , D. Riman , V. Halouzka , T. Opletal , D. Jirovsky , M. Prodromidis , J. Hrbac , J. Electroanal. Chem. 2018, 816, 45.

[52] D. Siegmund , S. Metz , V. Peinecke , T. E. Warner , C. Cremers , A. Grevé , T. Smolinka , D. Segets , U.‐P. Apfel , JACS Au 2021, 1, 527. 34467315 10.1021/jacsau.1c00092 PMC8395688

[53] M. E. G. Lyons , R. L. Doyle , I. Godwin , M. O'Brien , L. Russell , J. Electrochem. Soc. 2012, 159, H932.

[54] H. Bode , K. Dehmelt , J. Witte , Electrochim. Acta 1966, 11, 1079.

[55] E. S. Lambers , C. N. Dykstal , J. M. Seo , J. E. Rowe , Oxid. Met. 1996, 1996, 301.

[56] E. Fabbri , A. Habereder , K. Waltar , R. Kötz , T. J. Schmidt , Catal. Sci. Technol. 2014, 4, 3800.

[57] O. Enea , Electrochim. Acta 1990, 35, 375.

[58] L.‐F. Huang , M. J. Hutchison , R. J. Santucci , J. R. Scully , J. M. Rondinelli , J. Phys. Chem. C 2017, 121, 9782.

[59] R. Barnard , C. F. Randell , F. L. Tye , J. Appl. Electrochem. 1980, 10, 109.

[60] Z. P. Cano , M. G. Park , D. U. Lee , J. Fu , H. Liu , M. Fowler , Z. Chen , J. Phys. Chem. C 2018, 122, 20153.

[61] E. Iruin , Electrochim. Acta 2019, 320, 134557.

