# Supplementary material for: Investigation of Highly Active Carbon‐, Cobalt‐, and Noble Metal‐Free MnO2/NiO/Ni‐Based Bifunctional Air Electrodes for Metal–Air Batteries with an Alkaline Electrolyte
Source: Glob Chall. 2023 Apr 7;7(6):2200223. doi: 10.1002/gch2.202200223 (PMC10242538; doi:10.1002/gch2.202200223)
Supplement: Supplementary file 1 — Supporting Information [file GCH2-7-2200223-s001.pdf]

Global Challenges
Open Access

Supporting Information

for *Global Challenges.*, DOI 10.1002/gch2.202200223

Investigation of Highly Active Carbon-, Cobalt-, and Noble Metal-Free MnO_2/NiO/Ni-Based Bifunctional Air Electrodes for Metal–Air Batteries with an Alkaline Electrolyte

Marvin Kosin, Simon Dondrup, Jan Girschik, Jens Burfeind, Ulf-Peter Apfel and Anna Grevé**

Supporting Information

Investigation of highly active carbon-, cobalt-, and noble metal-free MnO_2/NiO/Ni-based bifunctional air electrodes for metal-air batteries with an alkaline electrolyte

Marvin Kosin, Simon Dondrup, Jan Girschik, Jens Burfeind, Ulf-Peter Apfel, and Anna Grevé**

Literature Research

The literature research to define the state-of-the-art was conducted via "ELib" (Fraunhofer Gesellschaft), using the following search string:

*(MnO2 OR "Manganese dioxide" OR "Manganese(IV) oxide") bifunctional (air OR oxygen) electrode**

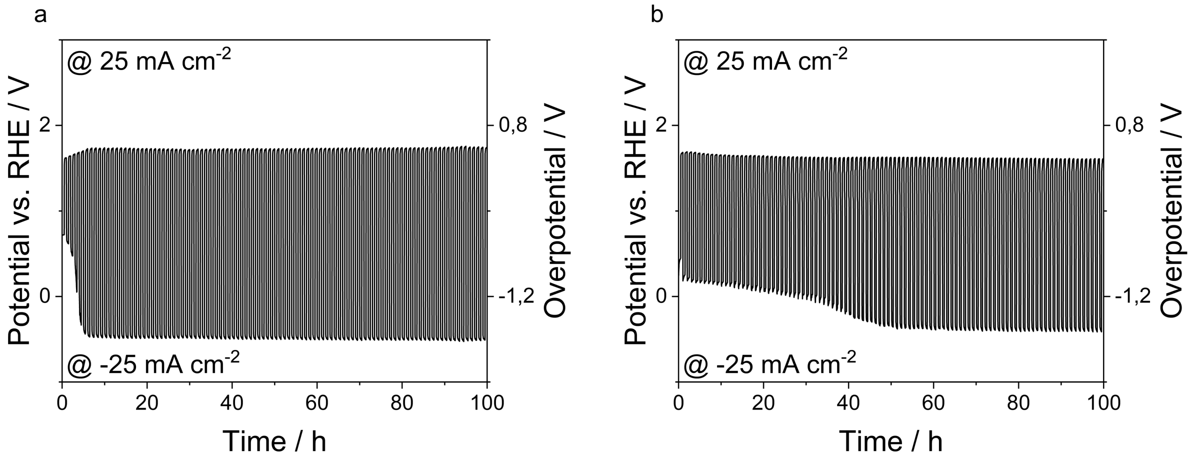

Figure S1. CP measurement of the MnO_2/Ni electrode (a) and the NiO/Ni electrode (b) with -25 mA cm^2 and 25 mA cm^2 for ORR and OER, respectively, applied for 30 minutes each.

Conversion of reference electrode potentials (Hg/HgO) to RHE

Potential vs. Hg/HgO / V	Potential vs. RHE / V
-0.6	0.324
-0.3	0.624
-0.2	0.724
-0.1	0.824
0	0.924
0.6	1.524
0.7	1.624
0.8	1.724

Used equations:

$$E \text{ vs. NHE} = E \text{ vs. Hg/HgO} + 0.098\text{ V} \quad \text{(S1)}$$

$$E \text{ vs. RHE} = E \text{ vs. NHE} + 0.059 * \text{pH} \quad \text{(S2)}$$

Reference: S. Niu, S. Li, Y. Du, X. Han, P. Xu, ACS Energy Lett. 5 (2020) 1083.

Conversion of reference electrode potentials (Zn/Zn^{2+}) to RHE

Potential vs. Zn/Zn^{2+} / V	Potential vs. RHE / V
0.92	0.983
1	1.063
1.1	1.163
1.15	1.213
1.93	1.993
2.04	2.103
2.1	2.163
2.25	2.313

Used equations:

$$E \text{ vs. NHE} = E \text{ vs. Zn/Zn}^{2+} - 0.763\text{ V} \quad \text{(S3)}$$

$$E \text{ vs. RHE} = E \text{ vs. NHE} + 0.059 * \text{pH} \quad \text{(S4)}$$

Reference: C.H. Hamann, W. Vielstich, Elektrochemie, Wiley-VCH-Verlag GmbH & Co. KGaA, Weinheim, 2005.

Detailed information about obtaining the electron transfer number

The electron transfer number for the ORR was determined according to the Koutecky-Levich equation, given by:

$$J^{-1} = J_K^{-1} + n^{-1}C\omega^{-0.5} \quad \text{(S5)}$$

n is the electron transfer number. C is a constant, which includes the Faraday constant, the viscosity of the electrolyte, the diffusivity of the reactants in the electrolyte, and the electrolyte concentration. The electron transfer number can be obtained from a variation of the rotating speed of the RDE and the slope of the resulting plot, as shown in Figure S2. To obtain the electron-transfer numbers, all other properties contributing to the constant C need to be known. The usual approach is to use literature values for the viscosity of the electrolyte, the diffusivity of the reactants in the electrolyte, and the electrolyte concentration. We used a different ansatz. Assuming that carbon catalyzes the two-electron pathway, we obtained C from this relation and calculated the electron transfer number for the catalyst samples according to:

$$n = 2C\left(\frac{dJ^{-1}}{d\omega^{-0.5}}\right)^{-1} \quad \text{(S6)}$$

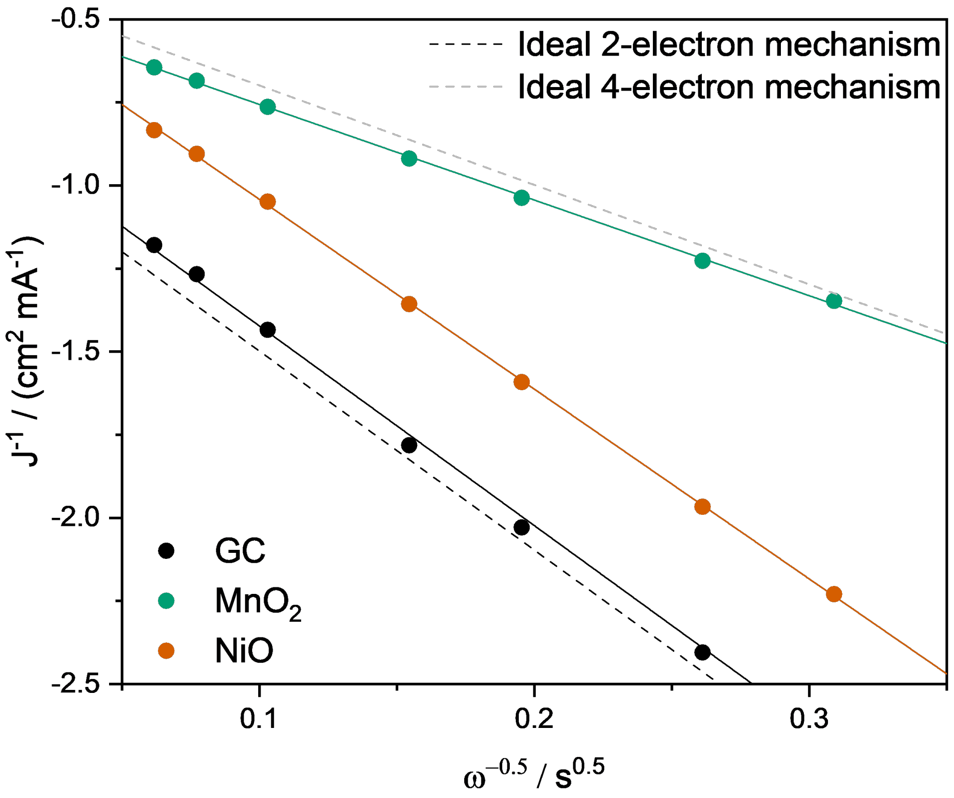

Figure S2. KL-plots obtained from LSVs at 0.4 V vs. RHE with a linear regression (Pearson R > 0.99 for all fits) and the theoretical slopes for an ideal two and four electron mechanism (c).

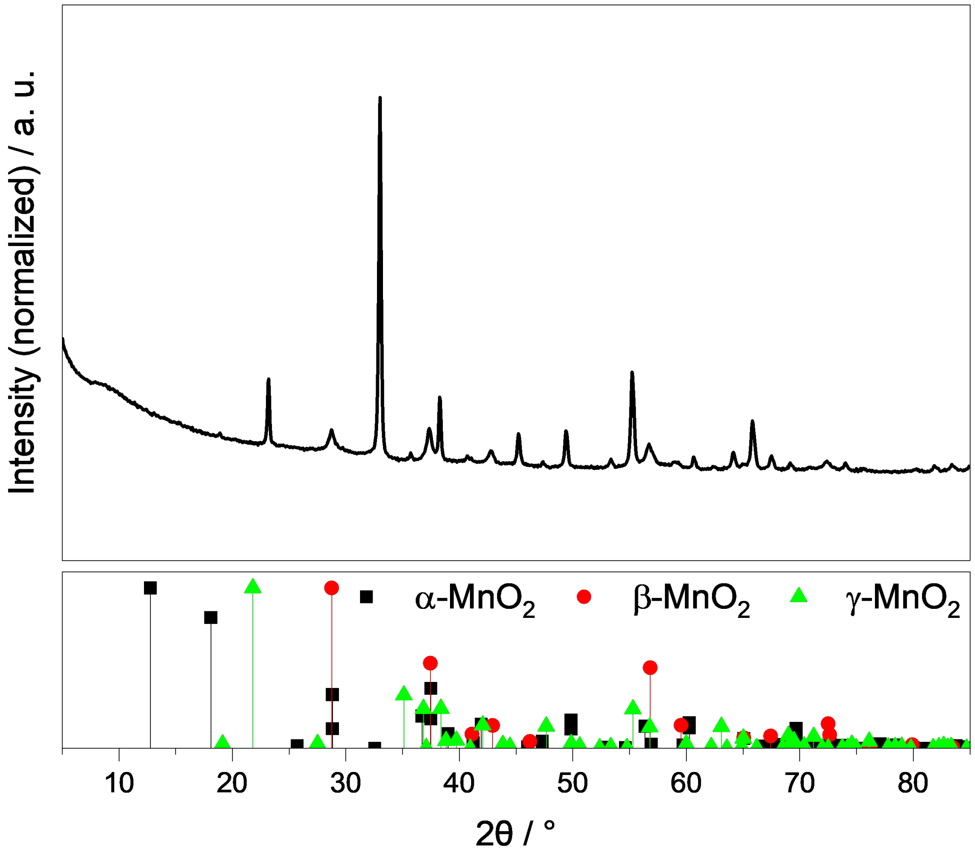

Figure S3. XRD spectrum of the commercial MnO_2 nanopowder (obtained from USRNM). The theoretical reflexes were calculated using VESTA. The corresponding CIF-files were obtained from the COD (IDs: 1514116, 1514101, and 1514102).

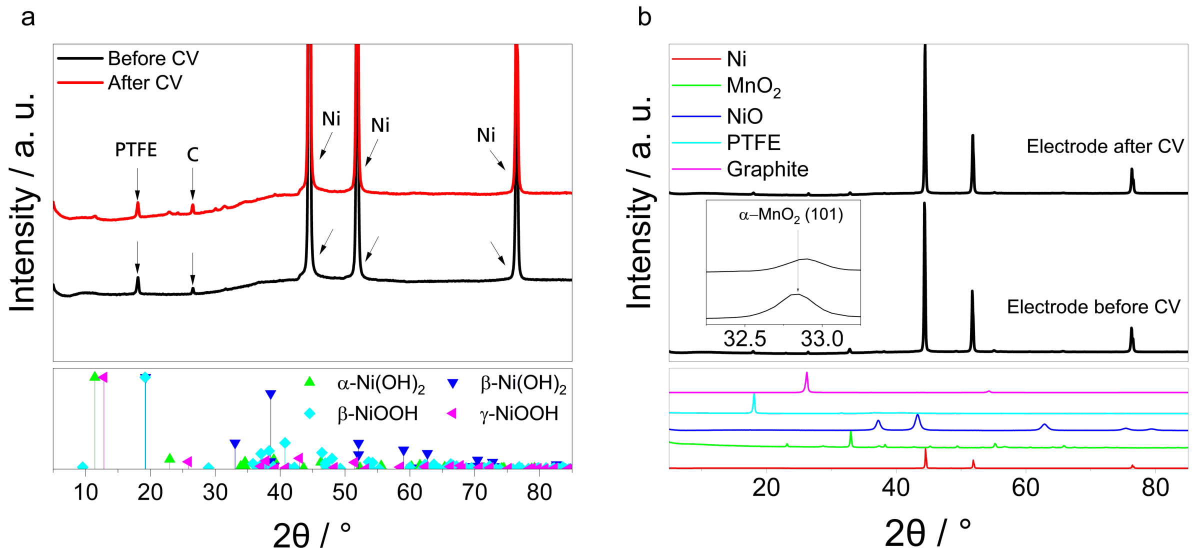

Figure S4. XRD spectra of the Ni bifunctional air electrode before and after CV-measurements (a). The references of the corresponding nickel (oxide) hydroxides are shown at the bottom of the plot. XRD spectra of MnO_2/Ni bifunctional air electrode before and after cycling (b) and the corresponding constituents (colored lines in the lower frame) of the electrode. Enlarged part of the spectrum with the corresponding reflex of the (101) plane of α-MnO_2 indicated. The peak that corresponds to graphite is attributed to remaining graphite of the plate that was placed on the electrode during hot pressing.

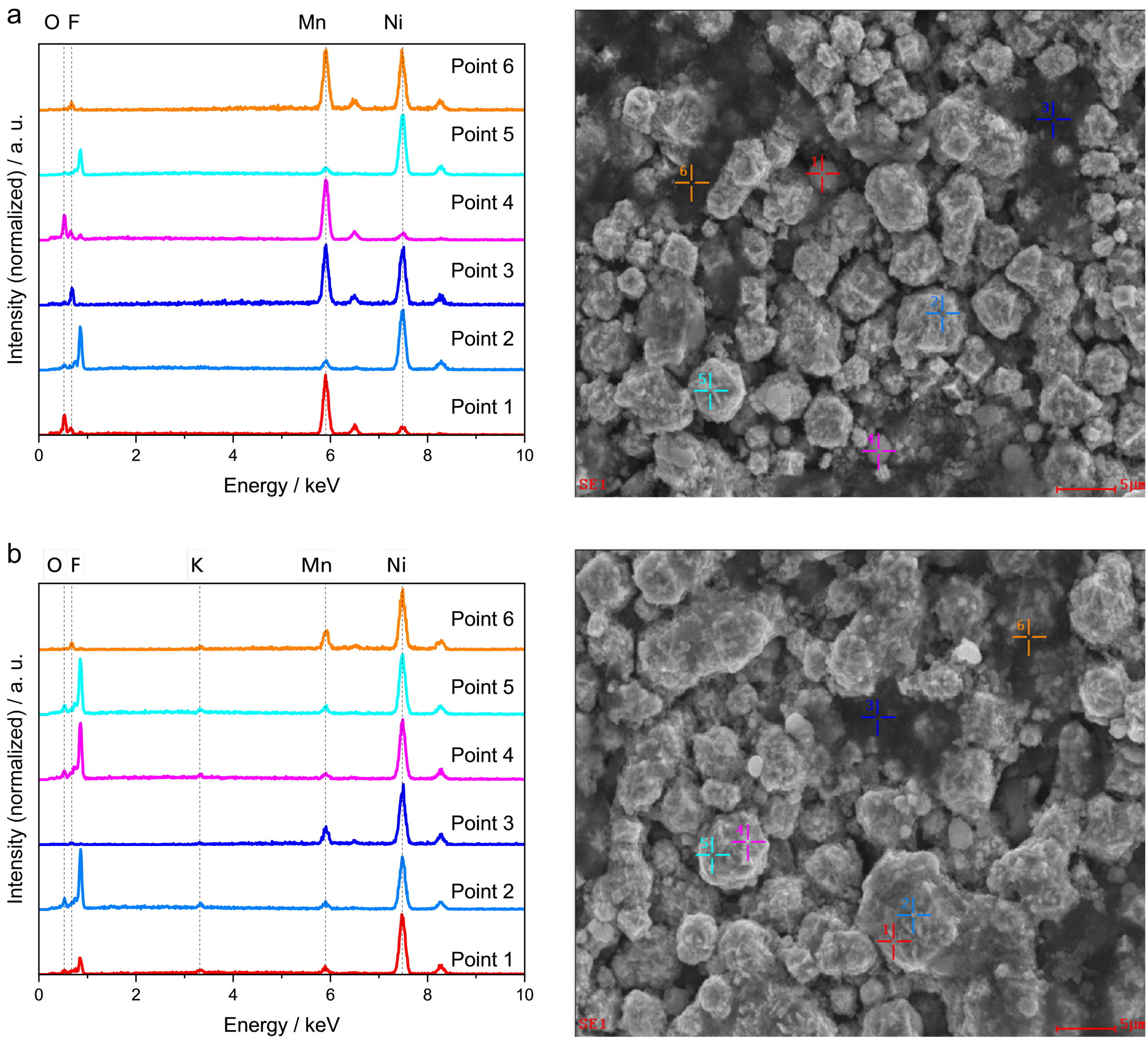

Figure S5. EDX spectra, performed at several points indicated within the SEM images of the MnO_2/Ni bifunctional air electrode before (a) and after (b) cycling.

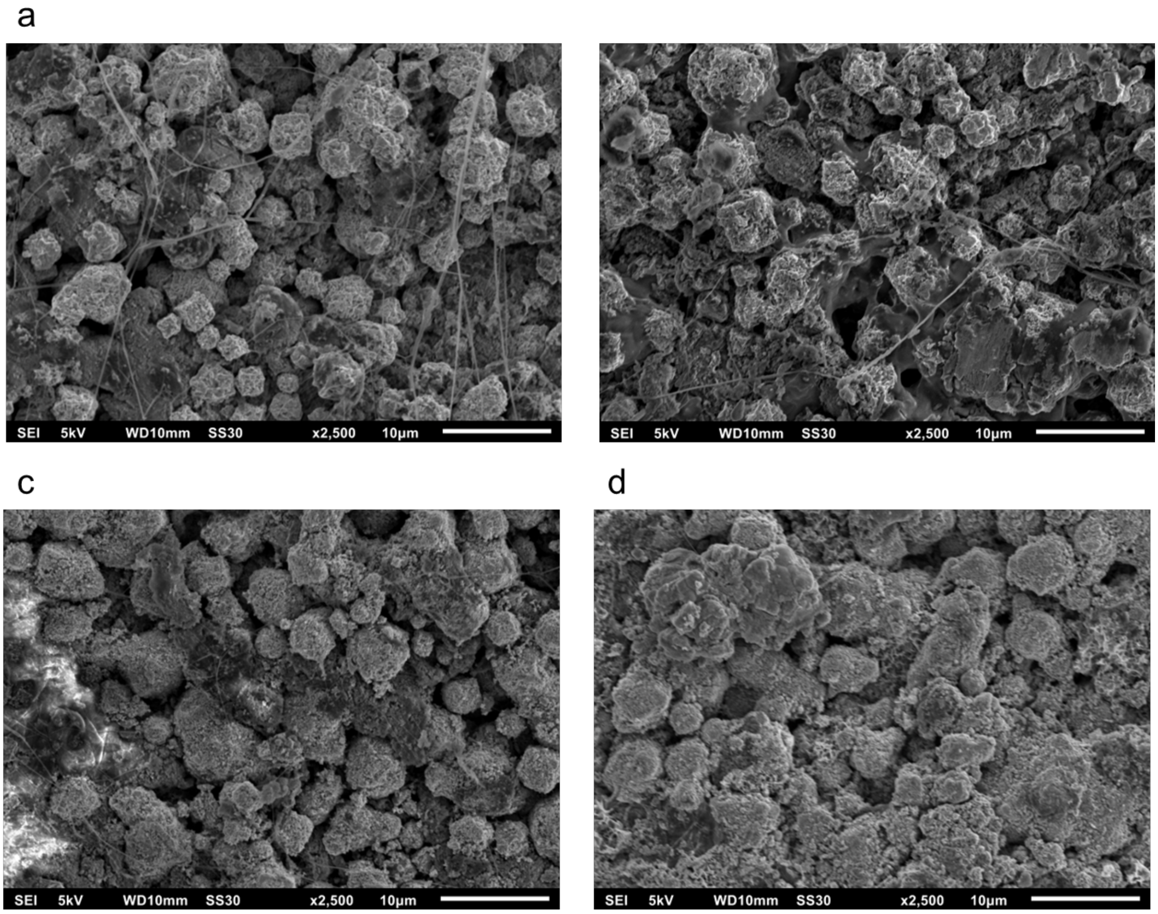

Figure S6. SEM images of the Ni and MnO_2/Ni bifunctional air electrode before (a, c) cycling and after (b, d) cycling at a magnification of 2500.
